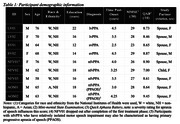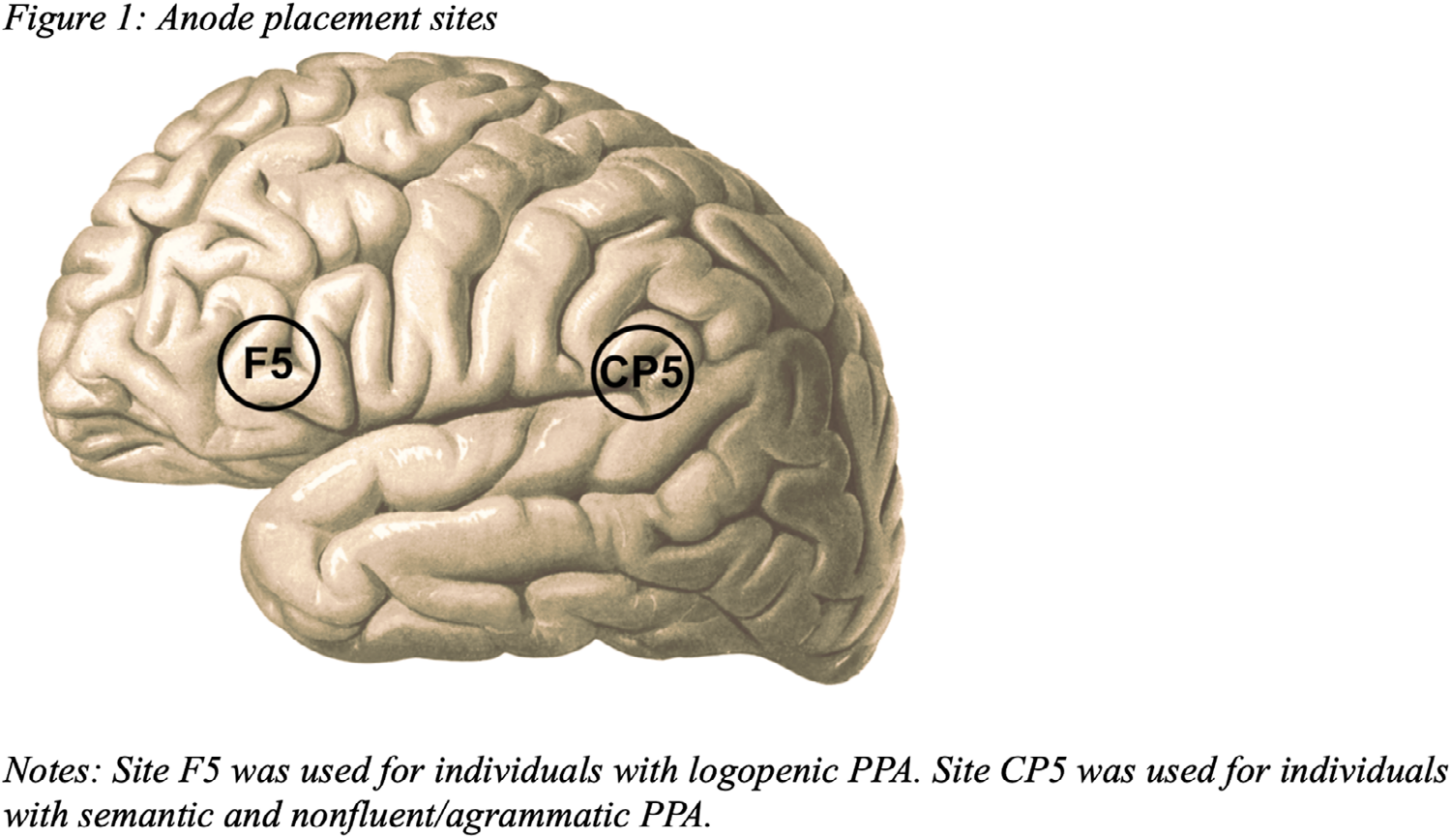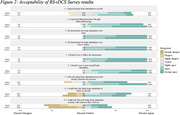# Combining script training and lexical retrieval treatment with remotely supervised transcranial direct current stimulation in primary progressive aphasia

**DOI:** 10.1002/alz70858_100323

**Published:** 2025-12-25

**Authors:** Lisa D. Wauters, Zoe Ezzes, Carly Millanski, David P. Baquirin, Alexia Hampson, Rachel Tessmer, Aakash Angirekula, Rian Bogley, Hannah Cho, Honey I. Hubbard, Sofia Fabi, Summer Fugere, Samantha Furnish, Buddhika Ratnasiri, Gary Robinaugh, Stephanie M Grasso, Maria Luisa Mandelli, Zachary Miller, Maria Luisa Gorno Tempini, Jessica D. Richardson, Maya L. Henry

**Affiliations:** ^1^ The University of Texas at Austin, Austin, TX, USA; ^2^ University of California San Francisco (UCSF), San Francisco, CA, USA; ^3^ Memory and Aging Center, UCSF Weill Institute for Neurosciences, University of California, Department of Neurology, San Francisco, CA, USA; ^4^ The University at Albany, Albany, NY, USA; ^5^ VA Pittsburgh Healthcare System, Pittsburgh, PA, USA; ^6^ Memory and Aging Center, UCSF Weill Institute for Neurosciences, University of California, San Francisco, San Francisco, CA, USA; ^7^ University of Southern California, Los Angeles, CA, USA; ^8^ University of New Mexico, Albuquerque, NM, USA; ^9^ Drexel University College of Medicine, Philadelphia, PA, USA; ^10^ University of Northern Colorado, Greeley, CO, USA; ^11^ Memory and Aging Center, Department of Neurology, Weill Institute for Neurosciences, University of California, San Francisco, San Francisco, CA, USA; ^12^ Department of Neurology, Memory and Aging Center, University of California San Francisco, San Francisco, CA, USA; ^13^ Dell Medical School at The University of Texas at Austin, Austin, TX, USA; ^14^ University of Texas at Austin, Austin, TX, USA

## Abstract

**Background:**

Transcranial direct current stimulation (tDCS) has shown promise as an adjunct to speech‐language treatment in primary progressive aphasia (PPA). However, the frequency of administration required for clinical trial protocols presents a barrier to participation for individuals who live in remote areas or have limited mobility. Additionally, though a handful of studies have delivered tDCS as an adjunct to treatments that address the core naming deficits associated with logopenic and semantic PPA variants (lvPPA, svPPA), very limited research has been completed that utilizes tDCS to address the core impairments associated with nonfluent/agrammatic variant PPA (nfvPPA; i.e., agrammatism and apraxia of speech). We piloted a trial protocol to address these limitations, delivering remotely supervised transcranial direct current stimulation (RS‐tDCS) in conjunction with established speech‐language tele‐rehabilitation approaches for each variant of primary progressive aphasia (PPA).

**Method:**

A sham‐controlled, double‐blind crossover design was implemented in which nine participants underwent lexical retrieval treatment (LRT; for lvPPA, svPPA) or video‐implemented script training for aphasia (VISTA; for nfvPPA) in conjunction with active or sham stimulation. Anodes were placed on left inferior frontal gyrus for logopenic PPA and temporoparietal cortex for semantic and nonfluent/agrammatic PPA. Cathodes were placed on the right shoulder. Data were collected to evaluate feasibility and acceptability of RS‐tDCS. Primary outcome measures for the interventions were proportion correctly named items (LRT) and proportion correct, intelligible scripted words (VISTA). Secondary outcome measures were linguistic and speech timing measures derived from connected speech as well as responses on the *Aphasia Impact Questionnaire* and *Communication Confidence Rating Scale for Aphasia*.

**Result:**

Results confirmed feasibility for all participants, and the trial protocol was considered acceptable. Results from the primary outcome measures indicated a robust response to treatment for all but one participant. Evidence of enhanced effectiveness in the active phase versus the sham phase varied among participants.

**Conclusion:**

Remotely‐delivered tDCS holds promise as a feasible and acceptable means to deliver accessible neuromodulation to individuals with PPA. Further work is needed to understand individual patterns of response to active versus sham stimulation conditions.